# Green nanogold activity in experimental breast carcinoma *in vivo*

**DOI:** 10.1042/BSR20200115

**Published:** 2020-11-25

**Authors:** Awatif A. Hendi, Doaa Mohamed El-Nagar, Manal A. Awad, Khalid M. Ortashi, Reema Abdullah Alnamlah, Nada M. Merghani

**Affiliations:** 1 Physics Department, College of Science, Princess Nourah Bint Abdul Rahman University, Riyadh, Saudi Arabia; 2 Faculty of Science and Humanities, Sattam University, Hota Bani Tamim, Saudi Arabia; 3 Zoology Department, College of Science,King Saud University, Riyadh 11421, Saudi Arabia; 4 Faculty of Women for Science, Arts and Education, Ain Shams University, Cairo, Egypt; 5 King Abdullah Institute for Nanotechnology, King Saud University, Riyadh 11451, Saudi Arabia; 6 Department of Chemical Engineering, King Saud University, Riyadh 11421, Saudi Arabia; 7 Central Lab and Prince Naif Centre for Heath Science Research, King Saud University,Riyadh 11421, Saudi Arabia

**Keywords:** breast cancer, Curcuma longa, DMBA, Green synthesis, NanoGold

## Abstract

**Background:** Over the past few years, fabrication of nanoparticles (NPs) has been deployed widely in technologies and many concerns have emerged about the hazardous effect on human health after NPs exposure.

**Objective:** Green synthesis of gold NPs (AuNPs) and assessment of their activity in 7,12-dimethylbenz(a)anthracene (DMBA)-induced breast cancer mouse model.

**Methods:** Chloroauric acid (HAuCl_4_) was used in formation of AuNPs with the help of *Curcuma longa* as aqueous reducing extract and stabilizing agent at room temperature. Formed NPs were characterized with UV-Vis spectrometry, Fourier-transform infrared spectroscopy (FTIR), Zetasizer measurement, Scanning Electron Microscopy (SEM) and Transmission Electron Microscopy (TEM). Virgin female albino mice with DMBA-induced breast cancer were treated with formed AuNPs for 5 consecutive days and were dissected after 28 days of the beginning of treatment.

**Results:** UV-Vis spectrometry showed absorbance maximum peak at 530 nm for formed AuNPs, FTIR confirmed formation of plant extract layer around formed NPs; zetasizer measurement revealed 278.2 nm as an average size of produced NPs; SEM and TEM approved formation of monodisperse spherical AuNPs. Biochemical analysis of untreated breast cancer group revealed marked changes in liver and kidney functions manifested by raised activity levels of alanine transaminase (ALT), aspartate aminotransferase (AST), blood urea nitrogen (BUN) and creatinine. Whereas, the treated group with AuNPs post-breast cancer induction displayed reduction in the activities (of ALT, AST and creatinine), while the BUN activity level was raised. Histopathological examination showed heavy incidence of tumor foci in the breast and lymph nodes belonged to the untreated breast cancer group confirmed with intense response to Ki-67 antibodies. While the treated group with AuNPs post-breast cancer induction showed degenerated tumor foci in the breast and lymph nodes with weak response to Ki-67 antibodies.

**Conclusion:** AuNPs were successfully synthesized using HAuCl_4_ and *C. longa* extract confirmed their ability to control DMBA-induced breast cancer in virgin female Swiss albino mice.

## Introduction

Research on nanotechnology and its applications is currently the most exciting area of focus in the field of materials science. As the conventional chemical and physical methods are expensive, several alternative methods are being used to synthesize nanoparticles (NPs) of silver, gold and other metal oxides. In addition to being expensive, the materials used in the preparation of NPs release harmful by-products, hence, low-cost and ecofriendly methods are being developed [1].

There are many physical and chemical methods for gold NPs (AuNPs) fabrication. The reduction of chloroauric acid (HAuCl_4_) by reducing agents such as hydrazine or sodium citrate is considered the most popular method. Whereas, the generated AuNPs are mostly not stable [2]. Alternatively, water-soluble polymers were used as reducing agents to produce more stable AuNPs as polyethyleneimine and poly(N-isopropyl-acrylamide-N,N-dimethylaminoethyl-acrylamide), their significance of reduction ability may be attributed to the amine functional group [3].

NP synthesis using plants, yeast, fungi, and bacteria is the new trend in nanotechnology, as it is economical, ecofriendly, and can be performed under ambient temperature, neutral pH, and by a single-step technique called green synthesis [4]. Combination of AuNPs with Ayurvedic phytochemical herbs as cinnamon, curcumin, grape, mango etc., led to innovative green nanoproducts called Nano-Ayurvedic formulations that will be a promising nanotechnology for cancer treatment [5].

The effectiveness of nanogold (AuNPs) in tumor treatment depends on their physical properties, specifically, their ability to permeate and accumulate in the tumor cells [6]. It has been observed that AuNPs (∼6−200 nm) can enter the tumor tissue, accumulate, and then damage the tumor cells [7].

The breast cancer is the commonest type of cancers among women. The real reasons of breast cancer are not completely understood, whereas many factors are associated with the risk of breast cancer as age, family history, benign breast lump, exposure to estrogen, hormone medicine, and radiation [8]. 7,12-dimethylbenz(a)anthracene (DMBA) is a hydrocarbon used as a carcinogenic and immunosuppressive agent to induce tumors in rodents. DMBA promotes the production of prostaglandin E2 (PGE2) which in turn increases the incidence of mammary tumors, in addition to other carcinogenic properties as mutation and disturbance of cellular oxidant–antioxidant balance [9].

Over the past years, the hazardous side effects of chemotherapy and radiotherapy for breast cancer have increased to an extent that in many cases have led to death. In addition, the non-selectivity of chemotherapy and radiotherapy results in great toxicity and oxidative stress all over the body [10].

The world has turned to look into innovative therapy strategies with less side effects. Therefore, the present work was designed to create AuNPs by green synthesis and investigate its potential anticancer activities on the breast cancer-induced in Swiss virgin female albino mice.

## Materials and methods

### Materials

*Curcuma longa* was purchased from Riyadh, KSA. Chlorauric acid (HAuCl_4_) and the Whatman filter paper (25 μm pore size and 15 cm diameter) were purchased from Sigma–Aldrich (Poole, U.K.). Distilled water was used to prepare the solutions.

### Synthesis of green nanogold (AuNPs)

*C. longa* extract was prepared by adding 50 mg of its powdered form to 100 ml distilled water and stirring the mixture continuously at room temperature (25°C) for 4 h. The solution was filtered and the filtrate was used as a green reducer and stabilizer. Ten milliliters of extract (prepared in Step 1) was mixed with 100 ml of (10^−3^ M) HAuCl_4_ aqueous solution under continuous and vigorous stirring for 24 h at room temperature. The color of the mixture gradually turned light violet, indicating the formation of the AuNPs. The synthesized AuNPs were purified through centrifugation at 9000 rpm for 15 min and the obtained pellet was collected for characterization.

### Characterization of green AuNPs

The synthesized AuNPs were characterized by UV-Vis spectra recording using a PerkinElmer UV-visible spectrometer (Lambda 25, PerkinElmer, United Kingdom) in the spectral range of 190−1100 nm; Fourier transform infrared spectroscopy (FTIR) spectra were recorded using a single-beam spectrometer genesis series Nicolet in the range of 4000–400 cm^−1^; dynamic light scattering (DLS) was used to measure the size of formed NPs using a Zetasizer, Nano series, HT Laser, ZEN 3600 (Malvern Instruments, U.K.) Thermo Scientific, Nicolet 6700; scanning electron microscopy (SEM) was performed using a JEOL-FE SEM to study the morphology and shape of AuNPs, and transmission electron microscopy (TEM) was performed using a JEM-1011, (JEOL, Japan) to characterize the size, shape, and morphology of the formed biogenic synthesized AuNPs. The samples were prepared by placing a drop of the solution containing the AuNPs on the carbon-coated TEM grids. The film on the TEM grid was allowed to dry at room temperature. Finally, energy-dispersive X-ray spectroscopy (EDS) analysis was performed to confirm the presence of gold in the particles as well as to detect other elementary components of the particles.

### Animals

The research was conducted using virgin female albino mice weighing 25 ± 5 g. The animals were collected from the University of Animal King Saud. Mice that have been handled in compliance with universal animal welfare laws. According to Prince Sattam University, the study was ethically accepted. At Sattam University Laboratories, all the experiments were carried out. They were kept in regular light and dark conditions and supplied with clean water and commercial food for mice.

### Breast cancer induction

A single dose (50 mg/kg) of DMBA, which was injected into the fat pad of the breast pairs, was given to virgin female mice. Three mice were randomly killed 10 days after the injection and subjected to histopathological analysis to confirm the induction of breast cancer.

### Experimental groups

Virgin female Swiss albino mice were divided into three groups of ten mice each: the first group acted as a control, while the second and third groups were subjected to breast cancer induction. The third group was treated with 100 µl/kg [11] of colloidal AuNPs for five consecutive days after cancer induction and left for 28 days after treatment. All mice were anesthesized by ether and killed at day 29.

### Breast index

After dissection, each mouse and its breast were weighed. The breast index was calculated by dividing the breast weight by the total body weight and multiplying by 100. The SPSS 16.0 software was used to calculate the mean and SEM.

### Biochemical analysis

Blood samples were collected from the hearts at day 29 and transferred into centrifuge tubes. Samples were centrifuged at 3000 rpm to separate the sera and stored at −80°C until assay. The sera were used to investigate the alanine transaminase (ALT), aspartate aminotransferase (AST), blood urea nitrogen (BUN), and creatinine levels of the specimens.

### Histological examination

The breast samples were fixed in 10% neutral buffered formalin, dehydrated, embedded in paraffin, and then cut into sections of 5-μm-thickness each. The sections were stained with Hematoxylin and Eosin stain (H&E), Masson’s Trichrome (M.Tr.), and then photographed with digital microscope (Motic-2000, China).

### Immunohistochemistry

The breast sections in the paraffin were mounted on coated slides and the paraffin was removed using xylene and descending grades of alcohol, followed by distilled water. These sections were then subjected to masking by citrate buffer (pH 6) in a microwave oven for 5 min. After washing with PBS buffer, the peroxidase blocking solution was drawn for 10 min. A primary antibody (anti-Ki-67) was used after overnight at 4°C followed by biotinylated goat anti-mouse as a secondary antibody incubation for 30 min. Subsequently, avidin–biotin complex was used for 30 min, and Diaminobenzidine (DAB) was used as a chromogenic substrate for 10 min. Finally, Mayer’s Hematoxylin was used as a counter stain and dehydration was performed through ascending grades of alcohol followed by xylene. All reagents were purchased from Abcam company (Cambridge, U.K.). The breast sections were examined and photographed at a magnification of 400×.

### Statistical analysis

The statistical significance of the control and experimental groups was evaluated using the SPSS 16.0 software, and the results compared; a value of *P*≤0.05 was considered significant.

## Results

### UV-Vis spectroscopy observation and analysis

In the present study, a new method was used for the synthesis of AuNPs from an aqueous HAuCl_4_ solution, using *C. longa* extract as a reducing, capping, and stabilizing agent. When the aqueous plant extract was mixed with aqueous HAuCl_4_ at room temperature, the color of the solution was observed to change in 24 h from pale yellow to purple, due to the formation of the AuNPs, the reason for this color change is the collective coherent oscillation of the conduction electrons at the surface of the AuNPs that interact with the oscillating electric field of the incident light; this phenomenon is called surface plasmon resonance (SPR). Hence, this change in color indicates the reduction in AuCl_4_^−^ ions, which was traced using UV-Vis spectroscopy*.*

**Figure 1:**
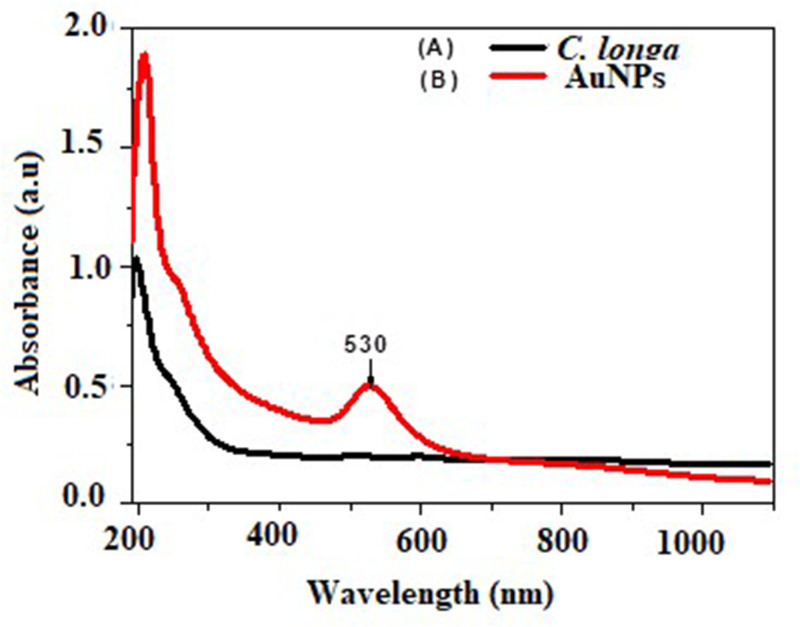
UV-Visible absorption spectra UV-Visible absorption spectra of (**A**) *C. longa* extract and (**B**) the green synthesized AuNPs showed its SPR bands at 530 nm.

Figure 1 shows the UV-Vis optical absorption spectra of the aqueous plant extract and the reduced HAuCl_4_ (colloidal solution of the AuNPs). These were recorded in the spectral range of 190−1100 nm with a maximum absorbance peak at ∼530 nm, which is the characteristic of monodispersed AuNPs with a little aggregation [12].

### FTIR spectroscopy

**Figure 2:**
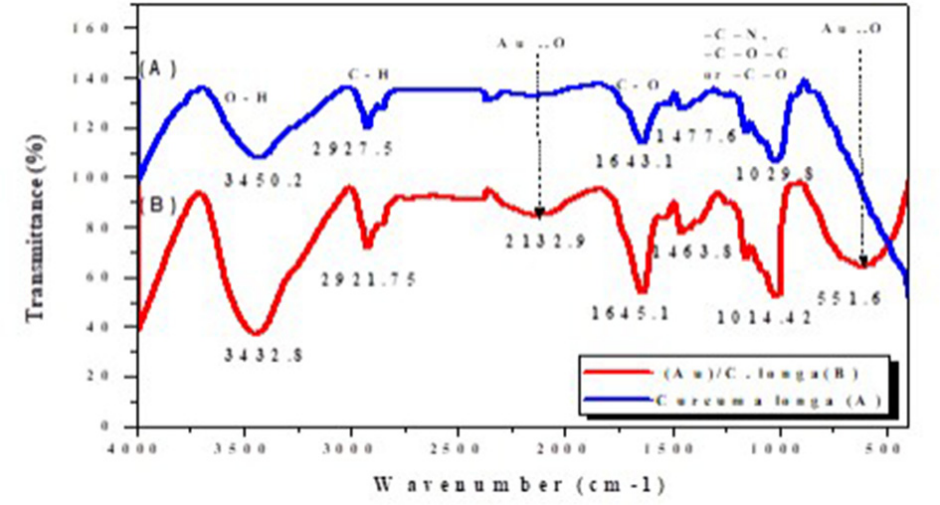
FTIR spectra FTIR spectra of (**A**) organic functional groups of *C. longa* extract and (**B**) their involvement attached to the surface of synthesized AuNPs. Figure 3, DLS analysis of the synthesized AuNPs.

FTIR is a chemical analytical technique that measures the infrared intensity versus wavelength (wavenumber) of light. It is used to determine the nature of the attentional molecules of plants or their extracts with NPs. Figure 2 shows the FTIR spectra of the *C. longa* aqueous extract and *C. longa* AuNPs.

The spectrum in Figure 2A shows transmission peaks at 3450.2, 2927.5, 1643.1, 1477.8, and 1029.8 cm^−1^. Similarly, the transmission peaks for AuNPs are observed at 3432.8, 2921.75, 1132.9, 1645.1, 1463.8, 1014.42, and 551.6 cm^−1^. The broad and strong bands at 3432.8 represent the –OH stretching vibrations, which confirm the presence of the hydroxyl groups (carbohydrates, flavonoids, and polyphenols) in the AuNPs. These hydroxyl groups lead to a negative charge around the AuNPs, which is evident from the negative ζ potential that is responsible for the stability of the *C. longa* AuNPs. The weaker band at 2921.75 cm^−1^ represents the –C–H stretching vibrations, due to the presence of the methyl, methoxy, and methylene groups. The peak at 1645.1 cm^−1^ is attributed to the carboxyl group (–C=O) stretching vibrations, which may be due to the presence of flavonoids, tannins, and terpenoids. The absorption at 1463.8 cm^−1^ indicates the presence of –NO_3_ in residual amounts. The absorption peaks at 1014.42 cm^−1^ can be attributed to the –C–N stretching vibrations of the amine, –C–O–C, or –C–O groups. Finally, the broad peaks at 551.6 and 2132.9 cm^−1^ are related to the AuNPs banding with the hydroxyl groups of the *C. longa* compounds (Figure 2B).

### Particle size determination by DLS

**Figure 3:**
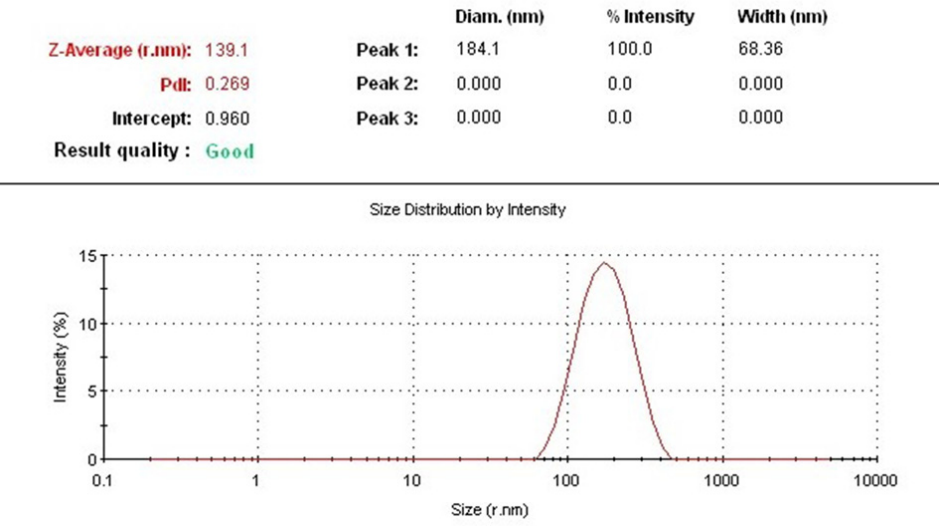
DLS analysis DLS measurement of the average size of synthesized AuNPs suspension.

DLS is a standardized technique that is based on the light scattering principle to measure particle size. This technique was used in the present study to determine the mean particle size and particle size distribution of AuNPs synthesized from an aqueous extract of *C. longa*. The average particle size of the AuNPs was 278.2 nm, which indicates medium size, as shown in the Figure 3. The average polydispersity index (PDI) of the synthesized AuNPs was 0.269. These results indicate monodispersity and stability of the synthesized NPs [13].

### SEM analysis and EDS analysis

**Figure 4:**
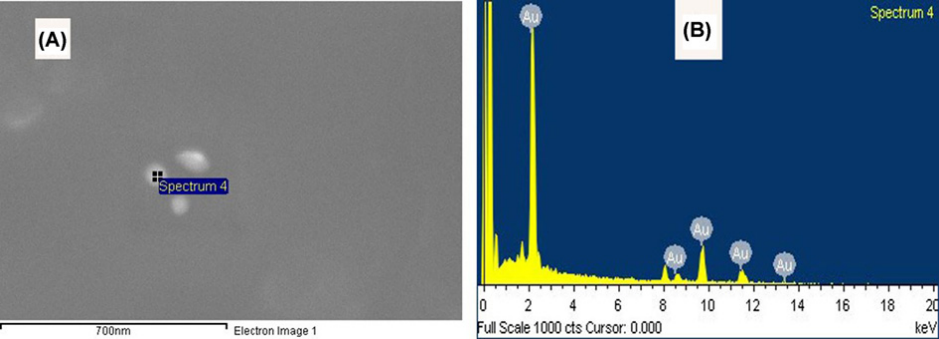
SEM images analysis (**A**) SEM image of synthesized AuNPs and (**B**) EDS analysis confirmed the formation of AuNPs

The scanning electron micrographs of the green AuNPs showed well-dispersed and spherical NPs (see Figure 4A). The EDS results confirmed the presence of the elemental signal of Au and the homogeneous distribution of the AuNPs. Furthermore, the EDS spectrum of the AuNPs showed strong peak at 2.2 keV (see Figure 4B).

### TEM analysis

**Figure 5:**
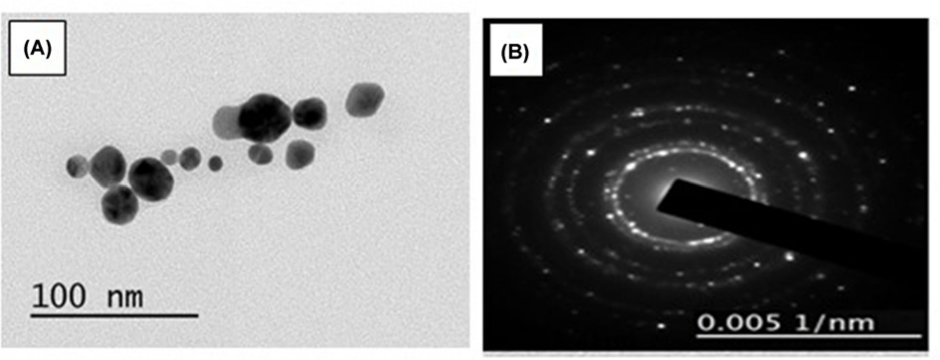
TEM images analysis (**A**) TEM image of the spherical shape and (**B**) SAED patteren of the green synthesized AuNPs.

The results of the TEM study revealed that most AuNPs prepared in the present study are spherical with variations in size (in the range of 10–30 nm), as shown in Figure 5A, which also indicates that they were distributed closely together. The results are in accordance with those of the UV-Vis spectroscopy. Furthermore, the selected-area electron diffraction (SAED) pattern indicates the crystalline nature of the AuNPs (Figure 5B).

### Breast index and biochemical analysis

Table 1 shows that the breast index of the cancer group is significantly higher than that of the control group. Whereas the cancer group treated with the AuNPs exhibits less breast index compared with untreated cancer group.

**Table 1:** Breast index and ALT, AST, BUN, and Creatinine levels of the investigated groups

Items	Control group	Breast cancer group	AuNP-treated breast cancer group
**Breast index**	0.2 ± 0.02	1.1 ± 0.2^1^	0.2 ± 0.02^2^
**ALT**	46 ± 0.5	77 ± 0.5^1^	47 ± 0.7^2^
**AST**	154 ± 0.5	167 ± 0.1^1^	155 ± 0.1^2^
**BUN**	20 ± 0.4	55 ± 0.3^1^	77 ± 1.5^1^^,^^2^
**Creatinine**	0.2 ± 0.4	0.4 ± 0.02	0.3 ± 0.04

Data = mean ± standard error of mean

1 , Significant difference compared with the control group.

2 , Significant difference compared with the breast cancer group.

Table 1 further shows that the ALT and AST levels of the cancer group are significantly higher than those of the control group. Whereas, the cancer group treated with the AuNPs exhibits levels that are significantly lower than the control group but significantly higher than those of the control group.

Furthermore, the BUN levels of the cancer group are significantly higher than those of the control group, while the cancer group treated with the AuNPs exhibits significantly higher levels than those of the control and cancer groups. Finally, the creatinine levels of the cancer group are insignificantly higher than those of the control group. Whereas cancer group treated with the AuNPs exhibits significantly higher levels than the control group.

### Histopathological analysis

**Figure 6:**
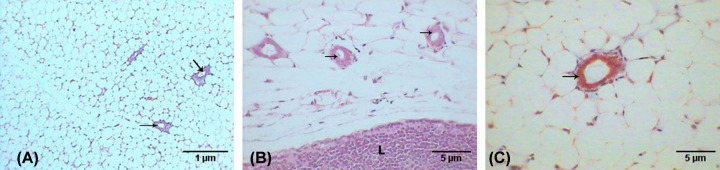
Photomicrographs of control breast tissue Photomicrographs showing (**A**) mammary glands (arrows) embedded in the adipose tissue (H&E: 100×), (**B**) mammary glands (arrows), lymph nodes (L) (H&E: 400×), (**C**) no fibrosis surrounded the mammary glands (M.Tr.: 400×).

The control breast, which showed the normal breast tissue of the virgin mice, revealed a few inactive glands with small ducts (Figure 6A). Moreover, the breasts are largely composed of adipose connective tissue in addition to presence of the lymph node, as shown in Figure 6B. In addition, control breast tissue showed no deposition of collagenous fibers as in Figure 6C.

**Figure 7:**
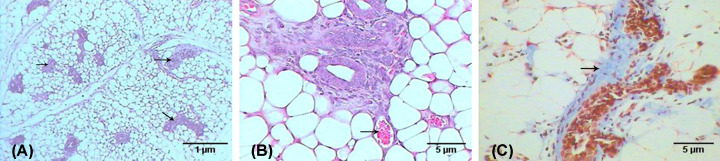
Photomicrographs of breast with tumor induced by DMBA Photomicrographs showing (**A**) multifoci of tumor nests (arrows) (H&E: 100×), (**B**) atypical tumor cells with hyperchromatic nuclei and newly formed blood vessel (arrow) (H&E: 400×), (**C**) desmoplastic reaction manifested by accumulation of collagenous fibers (arrow) around the tumor cells (M.Tr.: 400×).

A histopathological examination of the breast of the second group (untreated breast cancer group) showed multiple foci of tumor nests scattered in the breast tissue (see Figure 7A), which was mainly invasive ductal carcinoma, where atypical tumor cells form nests that induce desmoplastic reaction in stroma (abundant collagenous fibers). The tumor nest cells revealed marked pleomorphism (variation in size and shape) that failed to orient with respect to each other (anaplasia), in addition to nuclear irregularity, hyperchromasia, nuclear anaplasia, and angiogenesis (formation of new blood vessels in the tumor (see Figure 7B)). The dilated duct was abundantly filled with edema, and surrounded by fibers, scattered tumor cells, and some infiltrative cells (see Figure 7C).

**Figure 8:**
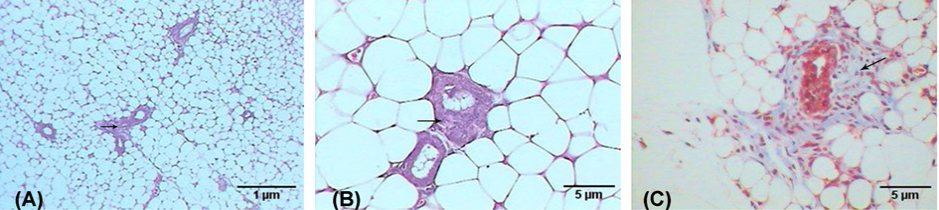
Photomicrographs of breast with tumor induced by DMBA and treated with Au NPs Photomicrographs showing (**A**) fewer tumor nests (arrows) (H&E: 100×), (**B**) small nest (arrow) (H&E: 400×), (**C**) lesser desmoplastic reaction of collagenous fibers (arrows) (M.Tr.: 400×).

In contrast, the breast examination of the third group (breast cancer group treated with AuNPs) revealed a marked improvement compared with the previous group, which is evidenced by the decreasing number of tumor foci in the adipose tissue of the breast (see Figure 8A), in addition to the minimized size of each nest (see Figure 8B), and the lesser desmoplastic reaction of the collagenous fibers (see Figure 8C).

**Figure 9:**
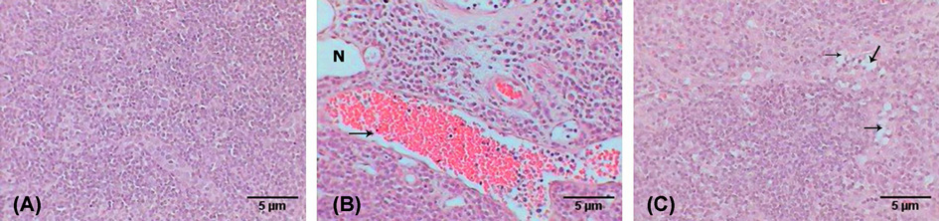
Photomicrographs of lymph node tissue Photomicrographs showing (**A**) normal structure of control lymph node, (**B**) lymph node after DMBA treatment revealing dilated and congested blood vessel (arrow), infiltrative cells, necrotic foci (N), (**C**) lymph node treated with AuNPs after DMBA treatment revealing small necrotic foci (arrows) (H&E: 400×).

The images of the control lymph node in Figure 9A show that the normal lymphoid tissue consists of lymphatic nodules that mostly contain B cells, T cells, and macrophages. In contrast, the breast cancer mice group revealed marked histopathological changes in the lymph nodes manifested by the presence of wide dilated, branched, and congested blood vessels (hemangiectasia), in addition to lymphocytic necrosis accompanied by the infiltration of inflammatory cell aggregations (see Figure 9B). However, the AuNP-treated breast cancer mice group exhibited considerable improvement compared with the breast cancer group, which is evidenced by the relatively healthy lymphoid tissue, although small necrotic foci can be observed, as shown in Figure 9C.

### Immunohistochemistry

**Figure 10:**
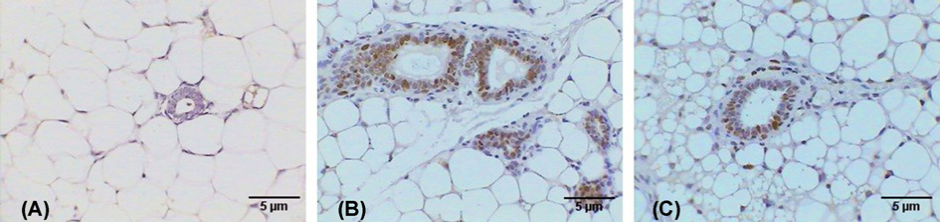
Photomicrographs of breast tissue stained immunohistochemically against Ki-67 antibodies Photomicrographs showing (**A**) no immune response in control, (**B**) the group of breast with tumor induced by DMBA displaying intense immune response in the tumor cells surrounded the mammary glands, (**C**) of breast with tumor induced by DMBA and treated with AuNPs revealing weak immune response (A–C: 400×).

The control breast tissue did not exhibit any immune response to the Ki-67 antibodies (Figure 10A), whereas the DMBA-induced cancer breast tissue exhibited many brown spots as an intense immune response to the Ki-67 antibodies. This indicates the high proliferation tendency of the cancer cells (Figure 10B). However, the AuNPs-treated cancer breast tissue displayed a few brown spots as a weak immune response to the Ki-67 antibodies indicating the low proliferation tendency of the cancer cells (Figure 10C).

**Figure 11:**
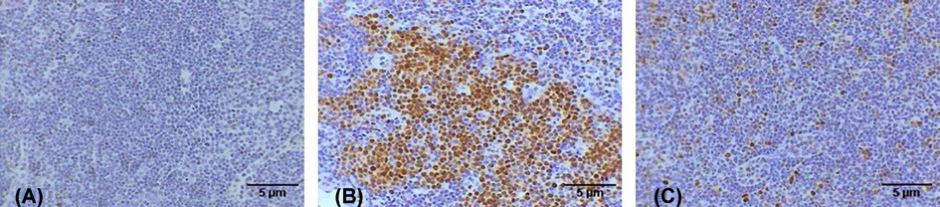
Photomicrographs of lymph node tissue stained immunohistochemically against Ki-67 antibodies Photomicrographs showing (**A**) no immune response in control, (**B**) lymph node after DMBA treatment revealing intense immune response, (**C**) lymph node treated with AuNPs after DMBA treatment displaying moderate immune response (A–C: 400×).

The control lymph node showed negative immunoreaction to Ki-67 (Figure 11A), the lymph node of the mice after breast cancer induction by DMBA showed intense immunoreactivity to Ki-67 antibodies manifested by the great aggregation of the cells stained dark brown (Figure 11B). However, the AuNPs-treated lymph node of the cancer group revealed several scattered dark brown cells as a weak immune response to the Ki-67 antibodies (Figure 11C).

## Discussion

In the present investigation, AuNPs were synthesized greenly using *C. longa* aqueous extract. A prerequisite for the application of the AuNPs in the treatment of cancer is that NPs must be produced in biological media supplies. Typically, the plant roots and leaves are an important source of antioxidant molecules such as polyphenols and flavonoids that have the ability to reduce metal ions. These antioxidant molecules can produce AuNPs by reducing gold ions (Au^3+^) [14]. It is known that the formation of AuNPs is indicated by the color change in the aqueous solution from yellow to purple, which occurs due to the excitation of the surface plasmons [15]; this is an intrinsic property of the AuNPs, and can hence be used to indicate their formation. The color of the AuNPs in the solution may vary from purple to dark purple with a characteristic absorption in the range of 520−560 nm, depending on their morphology, shape, and size [16,17]. The UV-Vis spectrum characterizes the green AuNPs synthesized from *C. longa* and monitoring the reduction of pure Au^3+^ ions into Au^0^. The combined vibration of the electrons of the metal NPs in resonance with the light waves released free electrons in the metal NPs, which causes the SPR absorption band observed at 530 nm. Similarly, Kumar et al. [18] have reported that an aqueous extract of the *Cassia auriculata* leaf exhibited SPR at 530 nm. The functional group responsible for the stabilization of the synthesized NPs was identified using FTIR measurement: the FTIR spectra of the AuNPs exhibited peaks at 3432.8 and 2921.75 cm^−1^, which correspond to the –OH stretching and aliphatic –C–H stretching vibrations [19]. The strong bands at 1645.1 cm^−1^ are attributed to the C=C and C=O stretching vibrations in the aromatic ring and polyphenols, respectively. The absorption bands located at 1463.8 and 1014.42 cm^−1^ may be attributed to the –C–O and –C–O–C stretching modes. The intense broad bands at 551.6 and 2132.9 cm^−1^ are attributed to the AuNPs banding from the hydroxyl groups. Thus, the FTIR results clearly show that the extracts contain −OH as a functional group, which function as the capping agents in the NP synthesis. This clearly establishes that the capping and reduction in the NPs by the biomolecules in the aqueous extracts of *C. longa* are responsible for the long-term stability; similar results were obtained by Noruzi et al. [20]. Furthermore, the results of the TEM, SAED, and SEM investigations helped determine the shape, size, and distribution of the NPs: the surface morphology of the NPs is crystalline spherical, and the size of the synthesized AuNPs is ∼10−30 nm [21]. The SEM result is proof that the morphology of the AuNPs is spherical and well-distributed without aggregation. This is in agreement with the data obtained from the TEM; similar results were obtained by Noruzi et al. [22]. The EDS result confirmed the existence and presence of typical metal gold nanocrystallite [23]. The particle size of the AuNPs was determined by DLS technique and was found to be 139.1 nm, which is larger than the size of the particles studied by TEM and SEM. This is because the particle size analyzer yield estimates of the hydrodynamic diameters of the NPs are different from the size obtained from the TEM and SEM studies; this could be due to the contrast between the AuNPs and the organic layers surrounding them, which is much larger, and is shown as larger particles when the particle size distribution is obtained using light scattering techniques [24].

Nanotechnology is considered as an innovative method in the field of medicine [25]. Especially, the AuNPs are relatively non-toxic and the body can get rid of these through the kidneys. Hainfeld et al. [26] reported smaller tumors and higher survival rate in mice injected with AuNPs (1−9 nm) compared with untreated control mice. Similarly, other studies proved reduction in the growth of the MDA-MB-23 breast cancer cell lines when treated with AuNPs accompanied with radiation [27]. Likewise, other studies have revealed that AuNPs might offer great promise for patients suffering from metastases after breast cancer [28]. Accordingly, the current results ensured marked improvement in the AuNPs-treated breast cancer group compared with the untreated breast cancer group represented by minimized tumor foci without congested blood vessels, leading to a normal breast index. The administration of DMBA increased the levels of AST and ALT in rats, which suggests DMBA-induced hepatic damage [29,30], which agrees well with the findings of the present study: induction of breast cancer by DMBA resulted in raised levels of ALT, AST, and BUN compared with the control group because of the hazardous effects of DMBA on the hepatic cells and kidneys. However, the present study also revealed that AuNPs-treated breast cancer group displayed normal levels of ALT and AST, indicating relatively healthy hepatic cells that agree with the findings of Jo et al. [31]. On the other hand, the observed higher BUN levels compared with the control and breast cancer groups may be due to the accumulation of the AuNPs in the kidneys. Moreover, Jain et al. [32] and Lee et al. [33] suggested that the AuNPs treatment dramatically reduced the tumor growth, which agrees with the current study: treatment with AuNPs after breast cancer induction showed reduction in tumor foci and desmoplastic reaction, alterations in the lymph nodes, and low proliferation tendency of the cancer cells in the breast and lymph nodes. Thus, the AuNPs have been demonstrated to easily permeate the tumor vasculature and remain in the tumors, leading to their prolonged existence, which increased their concentration, and finally resulted in damaging the tumor cells.

## Conclusion

Finally, it was concluded that the fabricated AuNPs by reduction of HAuCl4 by natural *C. longa* extract had impacted effect on breast cancer DMBA-induced in female Swiss albino mice, which reduced the incidence of tumor in the breast and lymph nodes; moreover, it improved the biological functions as liver and kidney functions that disturbed by tumor existence. Hence, the bioproduction of AuNPs still is a promising issue that needs more future investigations.

## Data Availability

The data will not be shared, because the identified participant information is included in the data.

## Abbreviations

ALT: alanine transaminase
AST: aspartate aminotransferase
AuNP: gold nanoparticle
BUN: blood urea nitrogen
DLS: dynamic light scattering
DMBA: 7,12-dimethylbenz(a)anthracene
EDS: energy-dispersive X-ray spectroscopy
FTIR: Fourier-transform infrared spectroscopy
HAuCl_4_: chloroauric acid
NP: nanoparticle
SAED: selected-area electron diffraction
SEM: Scanning Electron Microscopy
SPR: surface plasmon resonance
TEM: Transmission Electron Microscopy
